# Calcination‐Induced Pore Evolution in TiO_2_ Supports Governing Ni Accessibility for Dry Methane Reforming

**DOI:** 10.1002/open.202600011

**Published:** 2026-04-20

**Authors:** Sung‐Bin Choi, Ye‐Eun Jeon, Da‐Bin Kang, Min‐Young Kim, Chang Hyun Ko

**Affiliations:** ^1^ School of Chemical Engineering Chonnam National University Gwangju Republic of Korea

**Keywords:** dry reforming of methane, Ni accessibility, pore evolution, support calcination

## Abstract

The influence of support calcination temperature on pore evolution, nickel (Ni) accessibility, and catalytic behavior was investigated using Ni‐loaded titanium dioxide (TiO_2_) catalysts for the dry reforming of methane (DRM). The TiO_2_ supports calcined between 400°C and 1200°C showed a structural transition from a hierarchical mesoporous‐macroporous framework to predominantly macroporous and eventually plate‐like morphologies. Despite similar Ni loading (1.1–1.2 wt%), reducibility, and Ni particle size (∼60–86 nm), the accessibility of Ni sites varied significantly due to calcination‐induced structural evolution. Notably, the most active catalyst, Ni/TiO_2_‐HR‐C12, achieved a high initial CH_4_ conversion of ∼93% and an H_2_/CO ratio of ∼0.95 at 800°C. It also exhibited superior stability during 50 h at 700°C. Low‐temperature‐calcined support allowed Ni precursors to infiltrate mesopores, which collapsed during subsequent 900°C calcination, leading to Ni encapsulation and poor activity. In contrast, high‐temperature‐calcined supports (≥900°C) generated thermally stable structures that prevented Ni infiltration, preserving surface‐exposed Ni sites. This high site accessibility is a decisive factor governing the enhanced DRM performance. These findings demonstrate that support thermal stability and Ni accessibility outweigh mesoporosity or Ni particle size, offering insights for designing thermally robust oxide‐supported catalysts.

## Graphical abstract

Ni/TiO_2_ catalysts with tunable pore structures were developed to study how support calcination temperature affects Ni accessibility and dry reforming of methane (DRM, CH_4_ + CO_2_ → syngas). Low‐temperature TiO_2_ forms mesopores that trap Ni, while high‐temperature calcination generates macroporous/plate‐like structures that expose Ni and enhance catalytic performance.

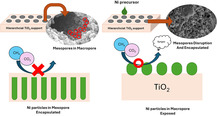

## 1 Introduction

Catalysts are widely employed in the chemical industry due to their ability to lower the activation energy of reactions, thereby enhancing reaction rates [1]. A typical heterogeneous catalyst consists of an active metal and support. While precious metals such as Pt, Pd, Ru, and Rh are effective, transition metals like Ni, Co, and Fe are often used due to their lower cost and sufficient activity. However, active metals used alone tend to suffer from limited dispersion and poor thermal or mechanical stability, necessitating the development of suitable support materials [2, 3, 4].

Supports such as SiO_2_, Al_2_O_3_, and TiO_2_ are commonly employed due to their low cost and tunable physicochemical properties. Among them, TiO_2_ is particularly attractive for catalytic applications not only because of its thermal stability but also due to its structural versatility [5, 6]. Recently, the “phase engineering” of catalyst supports, specifically controlling the crystalline phase to optimize C1 molecule conversion, has emerged as a critical strategy in catalyst design [7]. When synthesized via titanium isopropoxide (Ti(OiPr)_4_) hydrolysis, TiO_2_ can form a porous framework exhibiting both mesopores (2–50 nm) and macropores (>50 nm), yielding a hierarchical pore structure [8, 9, 10, 11]. These hierarchical supports are generally considered advantageous because they combine the high metal dispersion capabilities of mesopores with the efficient mass transport characteristics of macropores. Additionally, TiO_2_ undergoes anatase‐to‐rutile transformation at elevated temperatures, making rutile the thermodynamically stable phase under high‐temperature catalytic environments [12].

Rapidly increasing greenhouse gas emissions have intensified climate change, leading Intergovernmental Panel on Climate Change (IPCC) to emphasize the urgent need to achieve net‐zero emissions by 2050 [13]. As a potential solution, dry reforming of methane (DRM) has gained substantial attention for its ability to simultaneously utilize CH_4_ and CO_2_ to produce valuable syngas. Within the utilization pathway, significant efforts have focused on converting these C1 gases into value‐added chemicals through catalytic processes [14, 15, 16].

DRM is a highly endothermic reaction typically conducted at temperatures between 700°C and 1000°C [17]. While several studies have explored TiO_2_‐based catalysts by investigating preparation methods and Ni‐doping strategies, systematic research focusing on how the thermal history of TiO_2_ supports governs Ni accessibility remains relatively limited compared to Al_2_O_3_‐based systems [18, 19, 20]. Traditional strategies often prioritize maximizing specific surface area to ensure high Ni dispersion [21]. However, under harsh conditions, such hierarchical structures can undergo severe textural evolution and pore collapse through surface diffusion [12, 22]. Despite its importance, comprehensive guidelines for managing metal–support behavior specifically on TiO_2_ systems remain scarce [23, 24].

In this study, we investigate the governing role of TiO_2_ support calcination temperature in regulating hierarchical pore evolution and its subsequent impact on Ni accessibility for DRM. To clearly isolate the support effect, we maintained a low and consistent Ni loading (1.1–1.2 wt%), allowing us to observe how structural evolution dictates the fate of active sites independently of metal content. Our results reveal an unexpected trend: while the support calcined at 400°C initially provides the high porosity typically favored in catalysis, its structural instability leads to severe collapse during subsequent high‐temperature treatment, resulting in Ni encapsulation. In contrast, prestabilizing the support at higher temperatures ensures a thermally robust framework that keeps Ni sites surface‐exposed and catalytically accessible. This approach allowed us to achieve a CH_4_ conversion of ∼93% at 800°C despite the minimal surface area of the support. These findings shift the focus merely increasing initial surface area to ensuring thermal structural stability, offering a refined paradigm for designing robust catalysts for harsh, CO_2_‐based reforming environments.

## 2 Results and Discussion

### 2.1 Support Structural Evolution

**FIGURE 1:**
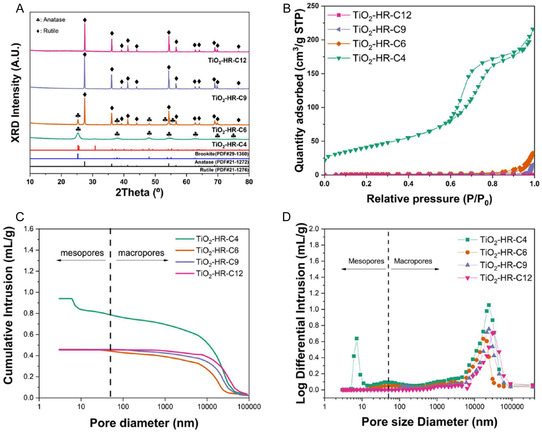
Structural and porosity evolution of TiO_2_‐HR‐C*X* supports. (A) XRD patterns illustrate the improvement in crystallinity and the phase transition from anatase to rutile as a function of calcination temperature. The symbols ♣ and ♦ identify the anatase (PDF#21−1272) and rutile (PDF#21−1276) phase TiO_2_. (B) N_2_ physisorption curves indicating progressive loss of mesoporosity. (C) Mercury intrusion profiles confirming retention of macropores but disappearance of mesopores at high temperature. (D) Log differential pore distributions illustrating mesopore collapse and structural densification.

The crystalline structure of the TiO_2_ supports evolved gradually with increasing calcination temperature, as confirmed by X‐ray diffraction (XRD) analysis (Figure 1A) [25]. For clarity, the samples are denoted as TiO_2_‐HR‐C*X*, where *X* represents the calcination temperature (TiO_2_‐HR‐C4: 400°C, TiO_2_‐HR‐C6: 600°C, TiO_2_‐HR‐C9: 900°C, TiO_2_‐HR‐C12: 1200°C). Phase assignment was conducted using standard reference patterns for anatase (PDF#21‐1272) and rutile (PDF#21‐1276). The TiO_2_‐HR‐C4 and TiO_2_‐HR‐C6 samples primarily exhibited the anatase phase (♣), with characteristic Bragg's angle clearly observed at 2*θ *= 25.3°, 37.8°, 48.0°, 53.9°, 55.1°, 62.7°, 70.3°, and 75.0°. As the calcination temperature increased to 900°C and above, a distinct phase transformation occurred. The TiO_2_‐HR‐C9 and TiO_2_‐HR‐C12 samples showed the dominance of the rutile phase (♦), identified by prominent peaks at 2*θ* = 27.4°, 36.1°, 39.2°, 41.2°, 44.1°, 54.3°, 56.6°, 62.7°, 64.0°, 69.0°, 69.8°, and 76.5° [26]. While brookite (PDF#29‐1360) was also considered as a reference, no characteristic peaks corresponding to this phase were detected in the experimental patterns. The TiO_2_‐HR‐C4 sample exhibited relatively broad peaks with low intensity, indicating low crystallinity. As the calcination temperature increased, the peak intensities for both anatase and rutile sharpened significantly, confirming the improvement in crystalline quality. Overall, the XRD results confirm that while calcination primarily enhanced crystallinity and induced the anatase to rutile transition, these changes were relatively minor compared with the pronounced modifications in the hierarchical pore structure described in the following section.

The N_2_ adsorption–desorption results showed that the overall adsorbed N_2_ gradually decreased with increasing calcination temperature, accompanied by a clear reduction in both BET surface area and total pore volume (Figure 1B and Table 1). The TiO_2_‐HRC4 and TiO_2_‐HR‐C6 supports exhibited distinct mesopore features, whereas the TiO_2_‐HR‐C9 and TiO_2_‐HR‐C12 samples showed negligible mesoporosity, indicating that the mesoporous network collapsed under high‐temperature calcination while the macropore framework was largely preserved [25, 27].

**TABLE 1:** BET surface area and total pore volume of TiO_2_‐HR‐C*X* supports showing progressive pore collapse with increasing calcination temperature.

	TiO_2_‐HR‐C4	TiO_2_‐HR‐C6	TiO_2_‐HR‐C9	TiO_2_‐HR‐C12
*S*_BET_ [m^2^ g^−1^]	150	4.48	1.40	0.20
Total pore volume (*P*/*P*_0_ = 0.990), cm^3 ^g^−1^	0.35	0.048	0.023	0.0006

**SCHEME 1:**
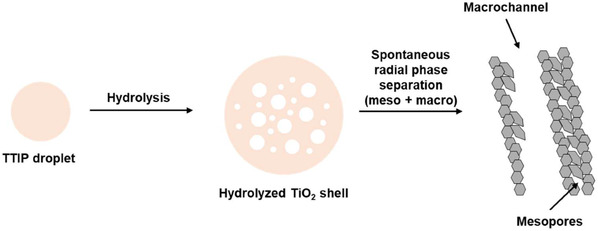
Proposed formation mechanism of hierarchical meso‐macroporous TiO_2_ during TTIP hydrolysis.

To further examine the pore structure, N_2_ physisorption and mercury intrusion porosimetry were performed together, allowing reliable characterization of both mesopores and macropores (Figure 1C,D) [28, 29]. All TiO_2_‐HR‐C*X* samples contained macropores, whereas mesopores were observed only in the low‐temperature calcined TiO_2_‐HR‐C4 and TiO_2_‐HR‐C6. The formation of this mesopore‐macropore network can be rationalized based on the hydrolysis behavior of titanium alkoxides in aqueous media. Upon contact with water, TTIP droplets rapidly form a thin, dense, semipermeable titania‐rich membrane at the droplet interface, which compartmentalizes subsequent hydrolysis and condensation reactions. As water diffuses inward through this membrane, hydrolysis proceeds from the outer shell toward the core, generating microphase‐separated domains consisting of TiO_2_‐rich regions and transient water/alcohol channels. The outward‐inward hydrolysis gradient and solvent hydrodynamics create rapidly patterned voids within each droplet, which evolve into mesopores after drying and low‐temperature calcination. In contrast, the larger fluid‐flow channels formed during the initial precipitation step persist as macropores (Scheme 1) [8, 9]. At higher calcination temperatures, the hydrated framework undergoes extensive sintering and structural rearrangement, leading to the collapse of mesopores while leaving the macropore framework largely intact.

**FIGURE 2:**
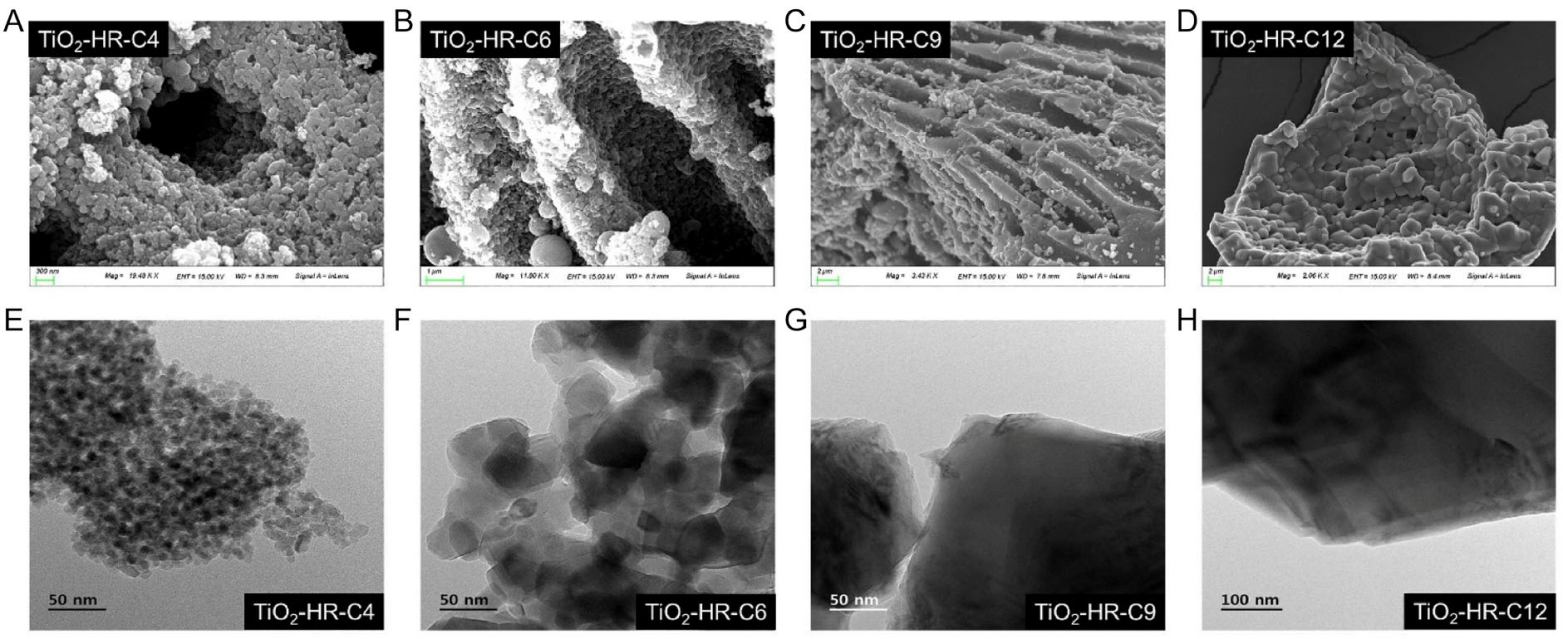
(A–D) SEM and (E–H) TEM images of the TiO_2_‐HR‐C*X* supports showing the effect of calcination temperature on pore morphology and structural evolution. TiO_2_‐HR‐C4 and TiO_2_‐HR‐C6 exhibit a hierarchical framework consisting of macropores with internal mesopores, whereas TiO_2_‐HR‐C9 and TiO_2_‐HR‐C12 show the disappearance of mesopores and the development of denser plate‐like domains, consistent with the textural and porosimetry results.

The SEM images revealed that the TiO_2_‐HR‐C4 and TiO_2_‐HR‐C6 supports possessed a macroporous network with pore sizes of ≈1.5 μm. In addition to the macroporous, mesopore on the scale of several tens of nanometers were observed within the pore walls (Figure 2A–D). The TiO_2_‐HR‐C6 support showed a densely interconnected morphology than TiO_2_‐HR‐C4, indicating that higher calcination temperature promoted further crystallization while still preserving the hierarchical pore system. In contrast, the TiO_2_‐HR‐C9 and TiO_2_‐HR‐C12 samples exhibited noticeably denser and more compact morphologies, with the macroporous framework remaining but the surface appearing smoother and more consolidated, consistent with sintering‐driven coalescence of primary TiO_2_ domains. These features suggest that the hierarchical structure transitions from a particle‐assembled framework at lower calcination temperatures to a more fused, grain‐like morphology at higher temperatures.

Transmission electron microscope (TEM) observations further supported these trends. (Figure 2E–H) TiO_2_‐HR‐C4 and TiO_2_‐HR‐C6 consisted of small, loosely aggregated TiO_2_ particles that contributed to the formation of internal mesopores. In contrast, TiO_2_‐HR‐C9 and TiO_2_‐HR‐C12 displayed a markedly different morphology in which the TiO_2_ structure evolved into extended sheet‐like domains rather than discrete particles. This morphological transition explains the disappearance of mesopores in the high‐temperature calcined samples, as the densification and lateral growth of the TiO_2_ domains eliminate the pore walls that originally sustained the mesoporous network. Consistent with these microscopic observations, TiO_2_‐HR‐C9 and TiO_2_‐HR‐C12 retained the overall macroporous framework but no longer displayed mesopores, in agreement with the N_2_ adsorption and mercury intrusion analysis that revealed collapse of the mesoporous network while the larger macropores remained stable. These results confirm that the hierarchical mesopore‐macropore structure is preserved only at lower calcination temperatures and progressively diminishes as thermal treatment induces structural rearrangement. Such structural transitions in the TiO_2_ supports are expected to influence how Ni species are distributed and exposed after impregnation and calcination, a relationship that will be examined in the following section.

### 2.2 Microstructural and Surface Characterization of Ni‐Loaded Catalysts

**FIGURE 3:**
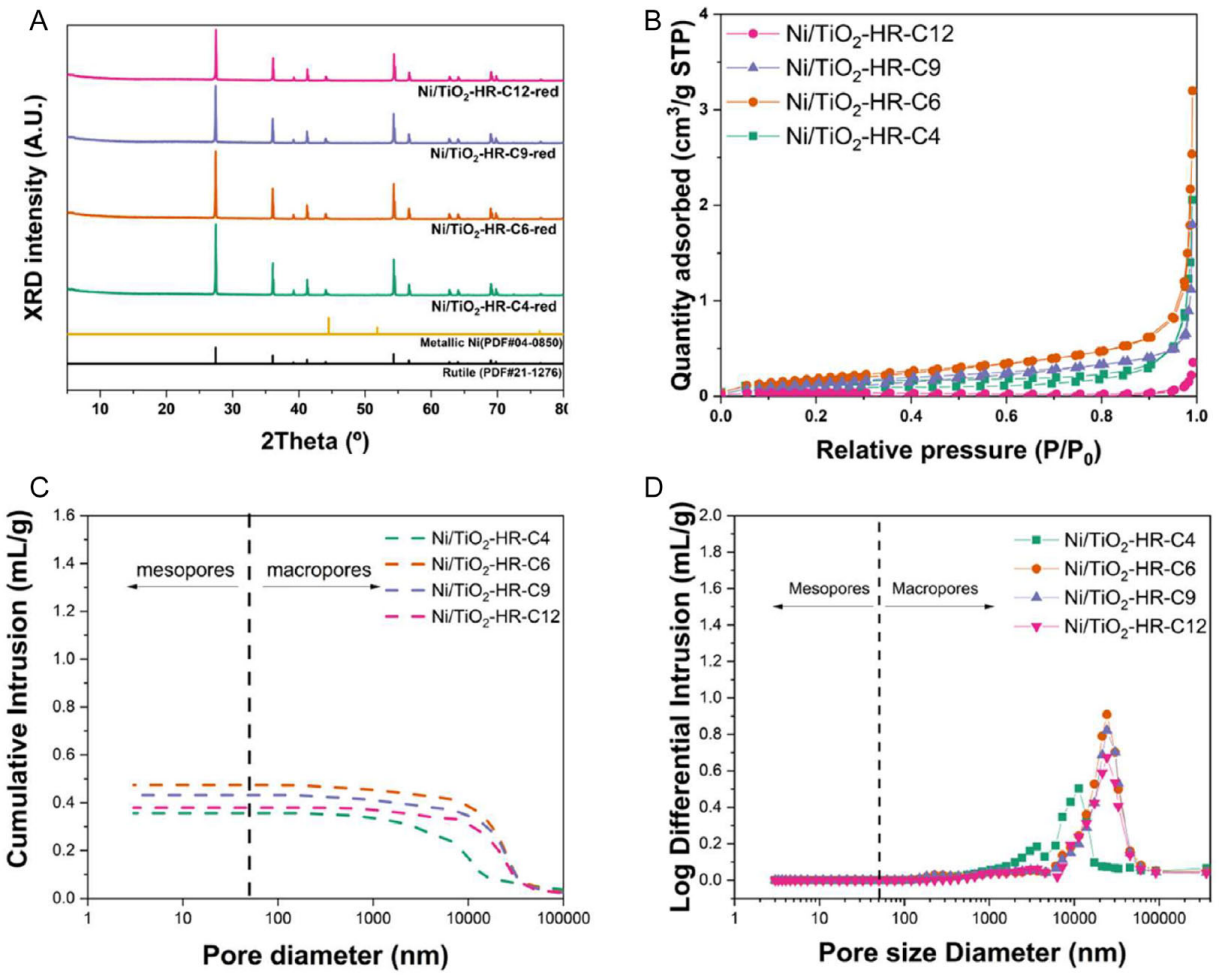
Structural and textural characteristics of Ni‐loaded TiO_2_‐HR‐C*X* catalysts. (A) XRD patterns recorded after Ni impregnation (calcined 900°C) and after H_2_ reduction at 800°C, showing the emergence of metallic Ni reflections (PDF#04‐0850) and the absence of detectable crystalline NiO (PDF#44‐1159). (B) N_2_ physisorption isotherms demonstrating reduced surface area and loss mesoporosity following Ni deposition and high‐temperature calcination. (C) Mercury intrusion profiles showing that macropores remain intact after Ni loading, while mesopores collapse in the low‐temperature‐derived supports. (D) Log‐differential pore‐size distributions confirming the disappearance of mesopores after Ni incorporation and the preservation of macropore frameworks across all catalysts.

To evaluate how Ni incorporation influences the structural and surface properties of the catalysts, a series of characterizations were conducted on the Ni/TiO_2_‐HR‐C*X* samples after impregnation, calcination, and reduction. The XRD patterns of the Ni/TiO_2_‐HR‐C*X* catalysts were examined to investigate changes in the nickel phase. After loading 1 wt% Ni and calcining the catalysts at 900°C, no distinct reflections attributable to NiO (PDF#44‐1159) were detected, likely due to the low Ni content and the high dispersion of Ni species on the TiO_2_ surface (Figure S1) [30]. In contrast, after reduction at 800°C under H_2_ flow, clear diffraction peaks corresponding to metallic Ni (PDF#04‐0850) emerged, indicating successful transformation of NiO or highly dispersed oxidized Ni species into metallic Ni (Figure 3A). The appearance of metallic Ni after reduction confirms that the active Ni phase was generated only upon H_2_ treatment, while the calcined catalysts remained predominantly TiO_2_ without detectable crystalline Ni‐containing phases.

Textural changes of TiO_2_ supports induced by Ni incorporation were also examined. The introduction of 1 wt% Ni followed by calcination at 900°C led to noticeable changes in the textural properties of the TiO_2_‐HR‐C*X* catalysts. After Ni impregnation, the BET surface area decreased for all samples, accompanied by a reduction in the total pore volume (Figure 3B and Table 2). This decrease in porosity likely reflects the collapse of fragile pore structures during high‐temperature calcination after Ni impregnation [31, 32]. As summarized in Table 2, the *S*_BET_ values of all final catalysts significantly decreased to below 0.8 m^2^/g after Ni loading and 900°C calcination, regardless of the initial support porosity (Figure 3C,D). This leveling of the specific surface area indicates that the fragile mesopores observed in the original TiO_2_‐HR‐C4 and C6 supports underwent extensive structural rearrangement and collapse during the high‐temperature treatment. Such physical consolidation of the support matrix is expected to significantly influence the final distribution and accessibility of Ni species, which will be further correlated with their catalytic performance in the following section [33].

**TABLE 2:** Textural properties, Ni loading, and Ni particle size of the Ni/TiO_2_‐HR‐C*X* catalysts after impregnation, calcination (900°C), and reduction (800°C).

	Ni/TiO_2_‐HR‐C4	Ni/TiO_2_‐HR‐C6	Ni/TiO_2_‐HR‐C9	Ni/TiO_2_‐HR‐C12
*S*_BET_, m^2 ^g^−1^	0.53	0.76	0.57	0.20
Total pore volume (*P*/*P*_0_ = 0.990), cm^3 ^g^−1^	0.0030	0.0042	0.0025	0.0006
Ni weight, %^a^	1.190	1.133	1.105	1.157
Average Ni particle size, nm^b^	67.63	71.74	59.85	85.93

a Ni content determined by ICP‐OES.

b Average Ni particle size estimated from TEM analysis (minimum 50 particles per sample) using Image J software.

Inductively coupled plasma optical emission spectroscopy (ICP‐OES) analysis verified that all Ni/TiO_2_‐HR‐C*X* catalysts contained nearly identical Ni loadings of ≈1.1–1.2 wt%, indicating that the amount of incorporated Ni remained consistent regardless of the support calcination temperature (Table 2).

**FIGURE 4:**
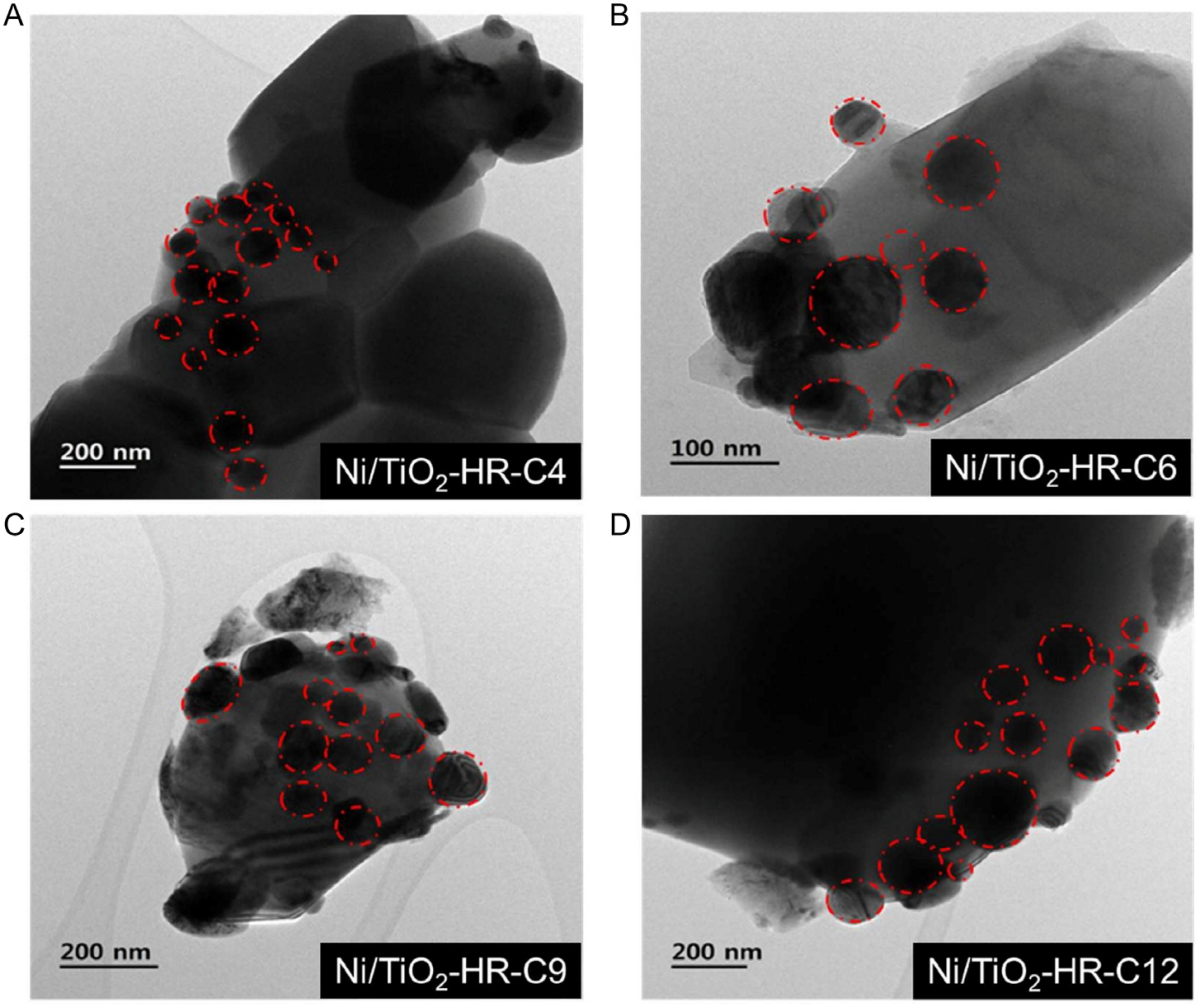
TEM images of the Ni/TiO_2_‐HR‐C*X* catalysts. (A) Ni/TiO_2_‐HR‐C4, (B) Ni/TiO_2_‐HR‐C6, (C) Ni/TiO_2_‐HR‐C9, and (D) Ni/TiO_2_‐HR‐C12. Representative Ni particles are marked with dashed red circles. All catalysts exhibit broadly distributed Ni particle sizes without a monotonic dependence on support calcination temperature. However, the extent of surface‐exposed Ni differs among samples, with Ni species more frequently located on external surfaces in the high‐temperature‐calcined Ni/TiO_2_‐HR‐C9 and Ni/TiO_2_‐HR‐C12 catalysts.

TEM analysis revealed broad Ni particle size distribution (average ≈60–86 nm) with no monotonic trend relative to the calcination temperature, indicating that support thermal history was not the primary determinant of Ni particle size (Figure 4A–D and Table 2) [34]. Despite the low Ni loading of 1 wt%, these relatively large particles resulted from the significant thermal densification of the TiO_2_ support during the final calcination at 900°C. The low specific surface area of the supports (<0.8 m^2^/g) led to high local surface density of Ni species, which facilitated extensive sintering and coalescence at high temperatures. Each catalyst exhibited a relatively large standard deviation, showing that Ni particles were dispersed over a wide size range regardless of the initial pore structure of the support. A direct quantification of Ni dispersion by H_2_ and CO chemisorption was attempted, but no measurable uptake was detected due to the very low Ni loading (1 wt%). Although pulsed N_2_O titration was also conducted, the results were physically inconsistent (e.g., calculated dispersion exceeding 100%), likely due to the inherent redox properties of the TiO_2_ support and nonspecific N_2_O consumption at the support surface, which obscures the titration of the minimal Ni active sites. Consequently, we instead utilized surface‐sensitive X‐ray photoelectron spectroscopy (XPS), CH_4_ temperature‐programmed surface reaction (CH_4_‐TPSR), and reaction kinetics as quantitative proxies for Ni accessibility. Therefore, chemisorption‐based dispersion analysis could not be applied to these catalysts [35]. However, the catalytic behavior was determined not by Ni particle size but by the location and accessibility of Ni species. For Ni/TiO_2_‐HR‐C4 and Ni/TiO_2_‐HR‐C6, the Ni precursor penetrated into the mesopores formed at low calcination temperatures, and these mesopores collapsed during the 900°C calcination, leading to partial encapsulation of Ni within the TiO_2_ matrix (Figure 4A,B). As a result, despite having similar particle sizes, these catalysts possessed fewer accessible Ni sites, yielding lower DRM activity. In contrast, Ni/TiO_2_‐HR‐C9 and Ni/TiO_2_‐HR‐C12 lacked mesopores, causing Ni species to remain on the external surfaces or macropore walls, which increased site accessibility and improved catalytic performance (Figure 4C,D).

Catalyst SEM images (Figure S2A–D) were also collected; however, the contrast between Ni and the TiO_2_ matrix was insufficient to distinguish the Ni dispersion reliably, and therefore the images are provided in Supporting Information for reference only.

**FIGURE 5:**
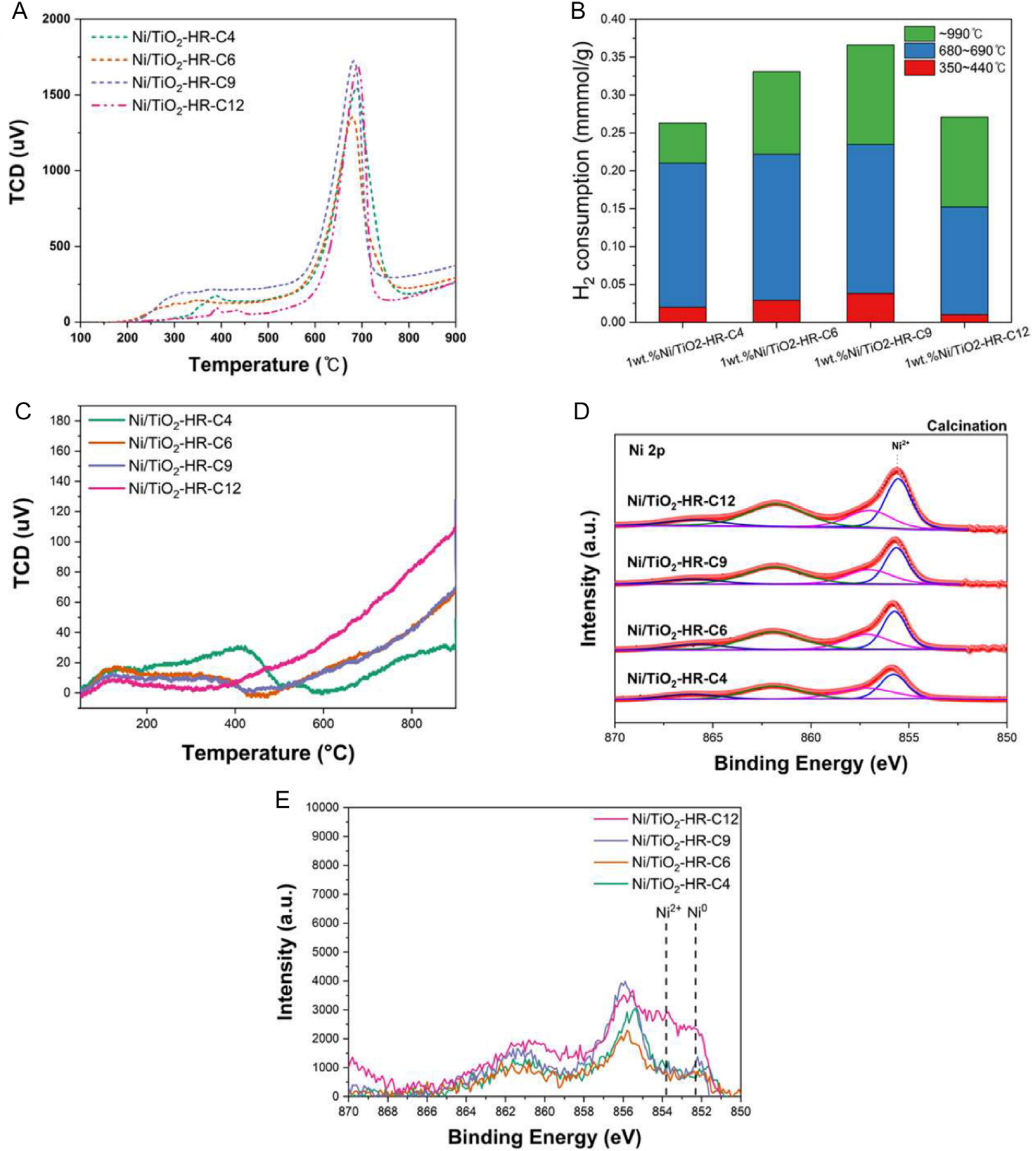
H_2_‐TPR and XPS analysis of Ni/TiO_2_‐HR‐C*X* catalysts. (A) H_2_‐TPR profiles showing similar behavior across the catalyst series, indicating that the reducibility of Ni species was not strongly affected by the calcination temperature of the TiO_2_ support. (B) Quantitative H_2_ consumption calculated from the TPR profiles. (C) CO_2_‐TPD profiles illustrating the density and strength of basic sites. (D) Ni 2p XPS spectra of calcined samples and (E) overlapped Ni 2p spectra after H_2_ reduction at 800°C. The surface Ni signals progressively increase from Ni/TiO_2_‐HR‐C4 to Ni/TiO_2_‐HR‐C12, demonstrating higher surface exposure of Ni species on the high‐temperature‐calcined supports, consistent with improved catalytic performance.

Hydrogen temperature‐programmed reduction (H_2_‐TPR) analysis revealed no significant variation in the reduction behavior of the Ni/TiO_2_‐HR‐CX catalysts with respect to the calcination temperature of the TiO_2_ support (Figure 5A). As summarized in Figure 5B, all samples exhibited three distinct reduction profiles: a minor peak at 350°C–440°C, a dominant peak centered at 680°C–690°C, and a high‐temperature tail near 1000°C. The predominant reduction peak observed around 690°C is attributed to Ni species in strong metal–support interaction with the TiO_2_ matrix. This high‐temperature feature suggests the presence of Ni species that are either strongly anchored to or partially encapsulated by the TiO_2_ framework, which is consistent with recent literature reporting robust Ni‐support interactions in this temperature range [36]. Quantitatively, the H_2_ consumption for this strongly anchored Ni‐support related peak was comparable across all catalysts (≈0.14–0.20 mmol/g), indicating that the chemical environment and the degree of Ni‐TiO_2_ interaction were largely independent of the initial pore structure of the support. The absence of distinct differences in Ni reducibility and the consistent presence of strong interaction reinforce that variations in DRM performance arise mainly from structural and accessibility differences rather than changes in the chemical environment of Ni.

The interaction between CO_2_ and the catalyst surface was evaluated using CO_2_‐TPD (Figure 5C). As shown in the profiles, the Ni/TiO_2_‐HR‐C4 catalyst exhibited a distinct desorption peak around 400°C, corresponding to medium‐strength basic sites. However, its CO_2_ interaction significantly diminished at higher temperature. In contrast, the prestabilized Ni/TiO_2_‐HR‐C12 catalyst showed a dominant and continuous increase in CO_2_ desorption above 600°C, reaching its maximum within the actual DRM reaction window (700°C–900°C). This suggests that the Ni/TiO_2_‐HR‐C12 catalyst possesses a higher density of thermally stable strong basic sites, which are beneficial for CO_2_ adsorption/activation and can contribute to the mitigation of coke formation under high‐temperature DRM conditions [37].

XPS analysis was performed to examine the surface chemical states and relative surface exposure of Ni species on the Ni/TiO_2_‐HR‐C*X* catalysts (Figure 5D,E). The spectra were calibrated using the C 1s peak at 284.8 eV, and the Ni 2p_3/2_ region was deconvoluted into Ni^2+^ (≈855.9 eV) and Ni^0^ (≈853.2 eV) [38, 39, 40]. As shown in the stacked spectra of the calcined catalysts (Figure 5D), the Ni 2p peak at ∼855.9 eV confirms that Ni exists primarily as Ni^2+^ (NiO) across all samples, indicating a consistent initial chemical environment after calcination. To clearly compare the relative surface exposure of the active Ni species after reduction, the spectra of the reduced catalysts overlapped in Figure 5E and stacked spectra in Figure S3A. This visualization reveals a nonlinear dependence of the surface Ni intensity on the support calcination temperature. While the high‐temperature stabilized catalysts (Ni/TiO_2_‐HR‐C9 and C12) displayed substantially higher intensities, a localized decrease was observed for Ni/TiO_2_‐HRC6. This deviation is attributed to the transitional nature of the support at 600°C; during the collapse of the hierarchical mesopore framework, a fraction of Ni species becomes temporarily trapped or shielded within the convoluted, partially collapsed pore boundaries. For the catalysts derived from Ni/TiO_2_‐HR‐C4 and Ni/TiO_2_‐HR‐C6, the XPS signals of Ni were markedly weaker. These results were closely related with the presence of mesopores in the low‐temperature calcined supports, which allowed Ni precursors to penetrate into the pore interior and become partially encapsulated upon the subsequent 900°C calcination. As a result, a significant portion of Ni species remained buried within the collapsed pore walls and were not detectable by surface‐sensitive XPS. Notably, the more severe deep encapsulation in the highly unstable Ni/TiO_2_‐HRC4 support leads to its lowest DRM performance, whereas Ni/TiO_2_‐HR‐C6 avoids such complete encapsulation due to partially trapped Ni species. In contrast, Ni/TiO_2_‐HR‐C9 and Ni/TiO_2_‐HR‐C12 lacked mesopores and exhibited sheet‐like morphologies, causing Ni species to remain primarily on the external surfaces on macropore walls. Consequently, these catalysts displayed substantially higher Ni^0^ and Ni^2+^ XPS intensities, confirming that Ni was more surface‐exposed and thus more accessible for catalytic activation. This trend correlates directly with the enhanced DRM performance observed for the high‐temperature calcined samples, highlighting the decisive role of Ni site accessibility rather than Ni particle size or reducibility. This structural interpretation is further corroborated by the XPS O 1s spectra (Figure S3B) and microscopic analysis. The progressive decrease in O 1s intensity with increasing support calcination temperature suggests a thermal densification and stabilization of the TiO_2_ framework. This stabilized surface acts as a robust platform that prevents Ni encapsulation, as suggested by the surface‐exposed Ni particles on the dense TiO_2_ plates in TEM images of the high‐temperature samples, in contrast to the more embedded Ni species within the textured clusters of the lower‐temperature supports.

### 2.3 DRM Reaction Performance Discussion

**FIGURE 6:**
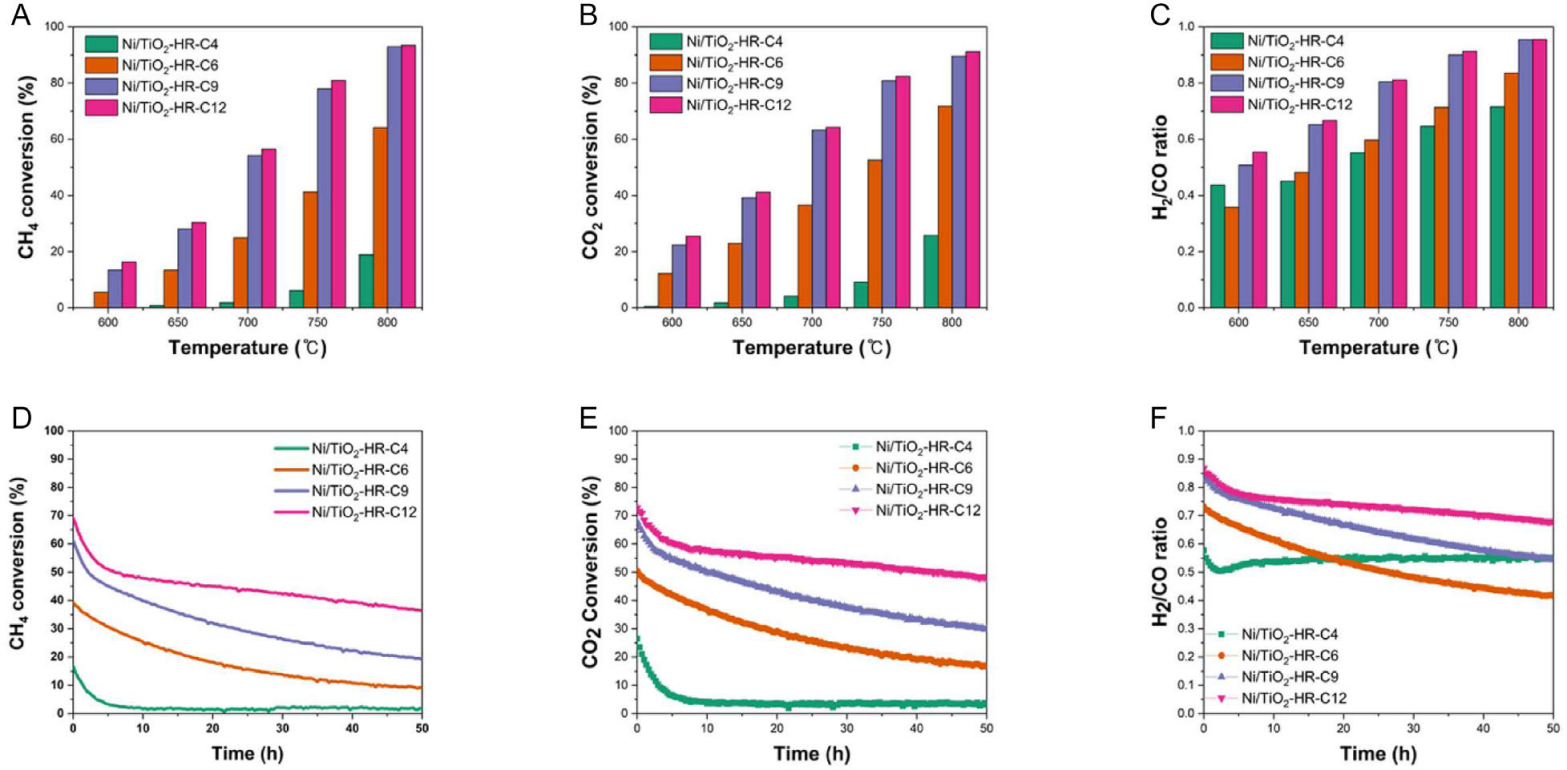
DRM performance of the Ni/TiO_2_‐HR‐C*X* catalysts. (A) CH_4_ conversion as a function of reaction temperature, showing progressively higher activity for catalysts prepared from high‐temperature‐calcined supports. (B) CO_2_ conversion under identical conditions, following the same trend as CH_4_ conversion and confirming increased reforming activity for Ni/TiO_2_‐HR‐C9 and Ni/TiO_2_‐HR‐C12. (C) H_2_/CO ratio plotted against temperature, approaching thermodynamic behavior for catalysts with higher surface‐exposed Ni. (D) Time‐on‐stream CH_4_ conversion at 700°C over 50 h, demonstrating significantly enhanced stability for Ni/TiO_2_‐HR‐C12 and Ni/TiO_2_‐HR‐C9. (E) CO_2_ conversion over 50 h, showing a similar stability trend consistent with CH_4_ conversion behavior. (F) H_2_/CO ratio during prolonged operation, indicating more stable syngas composition for high‐temperature‐calcined supports.

The catalytic performance of the Ni/TiO_2_‐HR‐C*X* series was first evaluated as a function of reaction temperature between 600°C and 800°C (Figure 6A–C and Figure S4A–C). A clear dependence on the calcination temperature of the TiO_2_ support was observed. Catalysts derived from high‐temperature‐calcined supports (TiO_2_‐HR‐C9 and TiO_2_‐HR‐C12) exhibited markedly higher methane conversion across the entire temperature range compared with TiO_2_‐HR‐C4 and TiO_2_‐HR‐C6 (Figure 6A). At 800°C, both Ni/TiO_2_‐HR‐C9 and Ni/TiO_2_‐HR‐C12 achieved initial CH_4_ conversions of ∼93%, with only a marginal difference between them. In contrast, Ni/TiO_2_‐HR‐C4 retained mesoporosity before Ni deposition yet showed much lower conversion (∼25%). A similar trend was also observed for CO_2_ conversion, confirming that the overall reforming rates followed the same structure‐dependent behavior (Figure 6B). The H_2_/CO ratio supported this trend: Ni/TiO_2_‐HR‐C9 and Ni/TiO_2_‐HR‐C12 approached equilibrium‐like values, whereas Ni/TiO_2_‐HR‐C4 exhibited an unexpected increase due to extremely low H_2_ formation that artificially inflated the ratio as CO dominated the syngas composition (Figure 6C). Collectively, these results demonstrate that DRM activity increases monotonically with support calcination temperature, highlighting the role of support evolution in governing the initial reactivity.

Long‐term stability was evaluated at 700°C for 50 h (Figure 6D–F). A consistent trend was observed: Ni/TiO_2_‐HR‐C12 maintained the highest CH_4_ conversion and exhibited the slowest deactivation, stabilizing at ∼40% after 50 h. Ni/TiO_2_‐HR‐C9 remained active, though its conversion declined to ∼20% over the same period (Figure 6D). In contrast, the catalysts derived from low‐temperature‐calcined supports showed significantly poorer stability; TiO_2_‐HR‐C6 retained only ∼10% conversion, while Ni/TiO_2_‐HR‐C4 became essentially inactive during the 50 h test. The H_2_/CO ratio also remained more stable for Ni/TiO_2_‐HR‐C9 and Ni/TiO_2_‐HR‐C12 during operation, whereas Ni/TiO_2_‐HR‐C4 again displayed unstable values due to insufficient H_2_ formation rather than improved syngas balance (Figure 6F). Consistent with the CH_4_ conversion trend, the CO_2_ conversion during the long‐term test also followed the same trend (Ni/TiO_2_‐HR‐C12 > Ni/TiO_2_‐HR‐C9 > Ni/TiO_2_‐HR‐C6 > Ni/TiO_2_‐HR‐C4), confirming that overall reforming behavior was strongly dependent on the structural evolution of the support (Figure 6E). These results indicate that higher calcination temperatures lead to structurally robust TiO_2_ frameworks that preserve Ni accessibility more effectively during extended high‐temperature operation. The reliability of these catalytic data was further validated by the carbon balance, which remained within 100 ± 2% for all tested catalysts throughout the reaction (Table S1 and Figure S5).

Despite similar Ni loadings (1.1–1.2 wt%), comparable Ni particle sizes (∼60–86 nm), and nearly identical reducibility profiles, the catalysts exhibited substantial differences in DRM reactivity and stability. Notably, although the *S*_BET_ values were leveled to a similar range across all catalysts (Table 2), a dramatic performance gap was observed (e.g., ∼25% for Ni/TiO_2_‐HR‐C4 vs. ∼93% for Ni/TiO_2_‐HR‐C12 at 800°C). Therefore, performance differences cannot be attributed to variations in Ni particle size, Ni specific surface area, Ni reducibility, or metal loading. Instead, the decisive factor is the accessibility of Ni species, governed by the structure of the TiO_2_ support.

For Ni/TiO_2_‐HR‐C4 and Ni/TiO_2_‐HR‐C6, the Ni precursor infiltrated the mesoporous network present in the low‐temperature‐calcined supports. Upon subsequent calcination at 900°C, these mesopores collapsed, leading to partial encapsulation of Ni within the TiO_2_ matrix. Although the overall Ni particle size after reduction was similar to that of Ni/TiO_2_‐HR‐C9 and Ni/TiO_2_‐HR‐C12, the fraction of surface‐exposed Ni available for CH_4_ and CO_2_ activation was significantly reduced. In contrast, Ni/TiO_2_‐HR‐C9 and Ni/TiO_2_‐HR‐C12 lacked mesopores and instead displayed macroporous plate‐like morphologies that remained stable during high‐temperature treatments. As a result, Ni species were preferentially deposited on external surfaces or macropore walls, preserving a much larger number of accessible active sites. This direct correlation between Ni accessibility and DRM performance explains why Ni/TiO_2_‐HR‐C9 and Ni/TiO_2_‐HR‐C12 show superior activity and stability relative to Ni/TiO_2_‐HR‐C4 and Ni/TiO_2_‐HR‐C6.

It is noteworthy that the superior DRM stability of Ni/TiO_2_‐HR‐C12, despite its significantly lower specific surface area, stems from a synergistic effect between structural stability and reaction‐relevant surface chemistry. While conventional catalyst design stratagy emphasizes maximizing initial porosity, our results suggest that such hierarchical structures are paradoxically vulnerable to phase‐transformation‐induced encapsulation under harsh conditions. In contrast, the prestabilized Ni/TiO_2_‐HR‐C12 provides a thermally robust rutile TiO_2_ support that not only preserves Ni accessibility but also maintains enhanced high‐temperature basicity (Figure 5C). This specialized surface chemistry facilitates efficient CO_2_ activation precisely within the reaction temperature window (700°C–900°C), ensuring the continuous gasification of surface carbon and thereby preventing the coke‐induced deactivation that plagues lower‐temperature counterparts.

**FIGURE 7:**
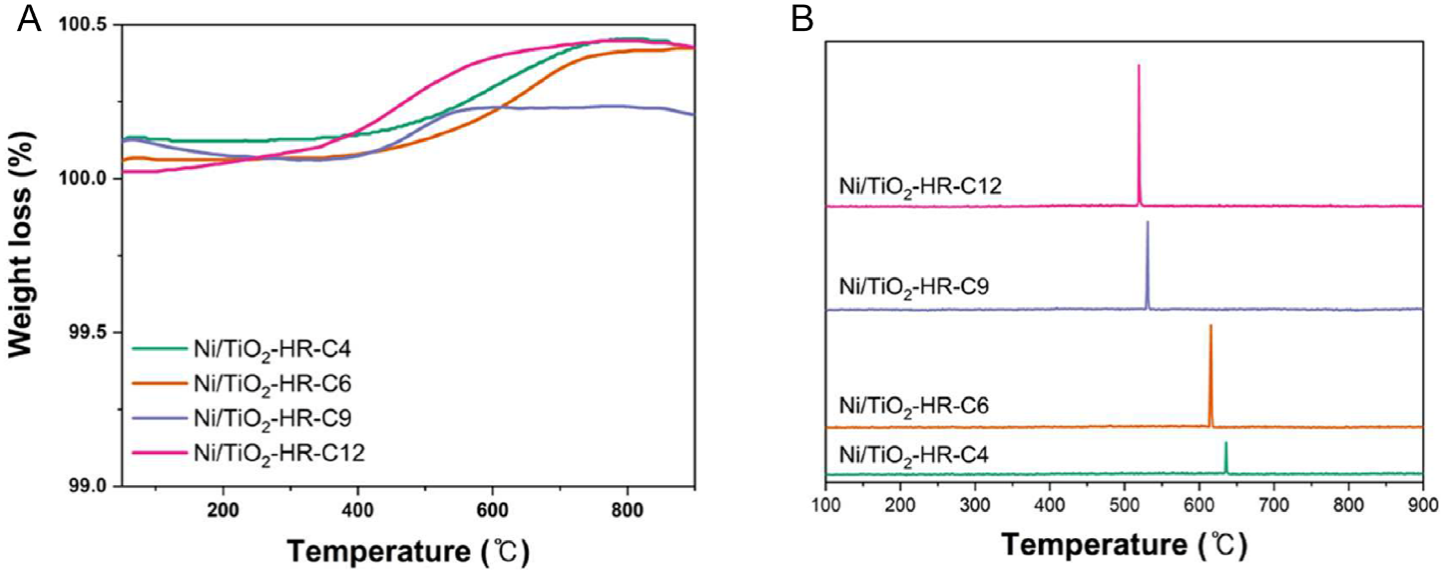
Coke quantification of Ni/TiO_2_‐HR‐C*X* catalysts after DRM. (A) TGA profiles showing no measurable carbon‐related mass gain, likely due to low coke accumulation and overlap with intrinsic TiO_2_ redox behavior. (B) TPO‐MS CO_2_ evolution profiles (*m*/*z* = 44) revealing relative coke amounts following the trend Ni/TiO_2_‐HR‐C4 < Ni/TiO_2_‐HR‐C6 ≈Ni/TiO_2_‐HR‐C9 < Ni/TiO_2_‐HR‐C12, consistent with activity‐dependent CH_4_ dissociation rather than Ni particle size or reducibility effects.

Coke formation was evaluated using thermogravimetric analysis (TGA) (Figure 7A) and TPO‐MS (Figure 7B). The TGA profiles of the spent catalysts under an air atmosphere primarily exhibited a net weight gain, which is attributed to the oxidation of metallic Ni to NiO. This weight gain dominated the overall profile because the amount of deposited carbon was relatively low, causing the weight gain from Ni oxidation to outweigh the weight loss from carbon combustion. Notably, Ni/TiO_2_‐HR‐C6 showed the minimum weight gain among the catalysts. This observation agrees with the XPS results (Figure 5E), where Ni/TiO_2_‐HR‐C6 exhibited the lowest surface Ni intensity due to the transitional shielding effect of the TiO_2_ support. The physical entrapment or shielding of Ni species within the convoluted pore boundaries of Ni/TiO_2_‐HR‐C6 not only limits its surface exposure but also hinders the accessibility of oxygen to the metallic Ni sites during TGA, thereby suppressing the extent of Ni oxidation. Therefore, TPO‐MS was employed to compare relative coke amounts across the catalyst series. The CO_2_ evolution profiles revealed the trend: Ni/TiO_2_‐HR‐C4 < Ni/TiO_2_‐HR‐C6 ≈Ni/TiO_2_‐HR‐C9 < Ni/TiO_2_‐HR‐C12. The negligible coke signal for Ni/TiO_2_‐HR‐C4 is consistent with its low catalytic activity, which limits CH_4_ dissociation and carbon‐forming pathways. For Ni/TiO_2_‐HR‐C6 and Ni/TiO_2_‐HR‐C9, moderate coke formation was observed, reflecting their intermediate activity levels. Notably, Ni/TiO_2_‐HR‐C12 exhibited the highest coke formation, despite also showing the highest activity and stability. This behavior reflects the dual effects of high Ni accessibility and slightly larger Ni domains: although these features enhance DRM activity, they also facilitate CH_4_ activation and carbon growth under locally oxygen‐deficient environments. Thus, coke formation follows the intrinsic CH_4_ activation capability of the catalysts and is modulated by subtle Ni size variations, but is ultimately governed by the accessibility of surface Ni sites provided by the thermally evolved TiO_2_ supports.

### 2.4 Surface Reactivity and Kinetic Interpretation

**FIGURE 8:**
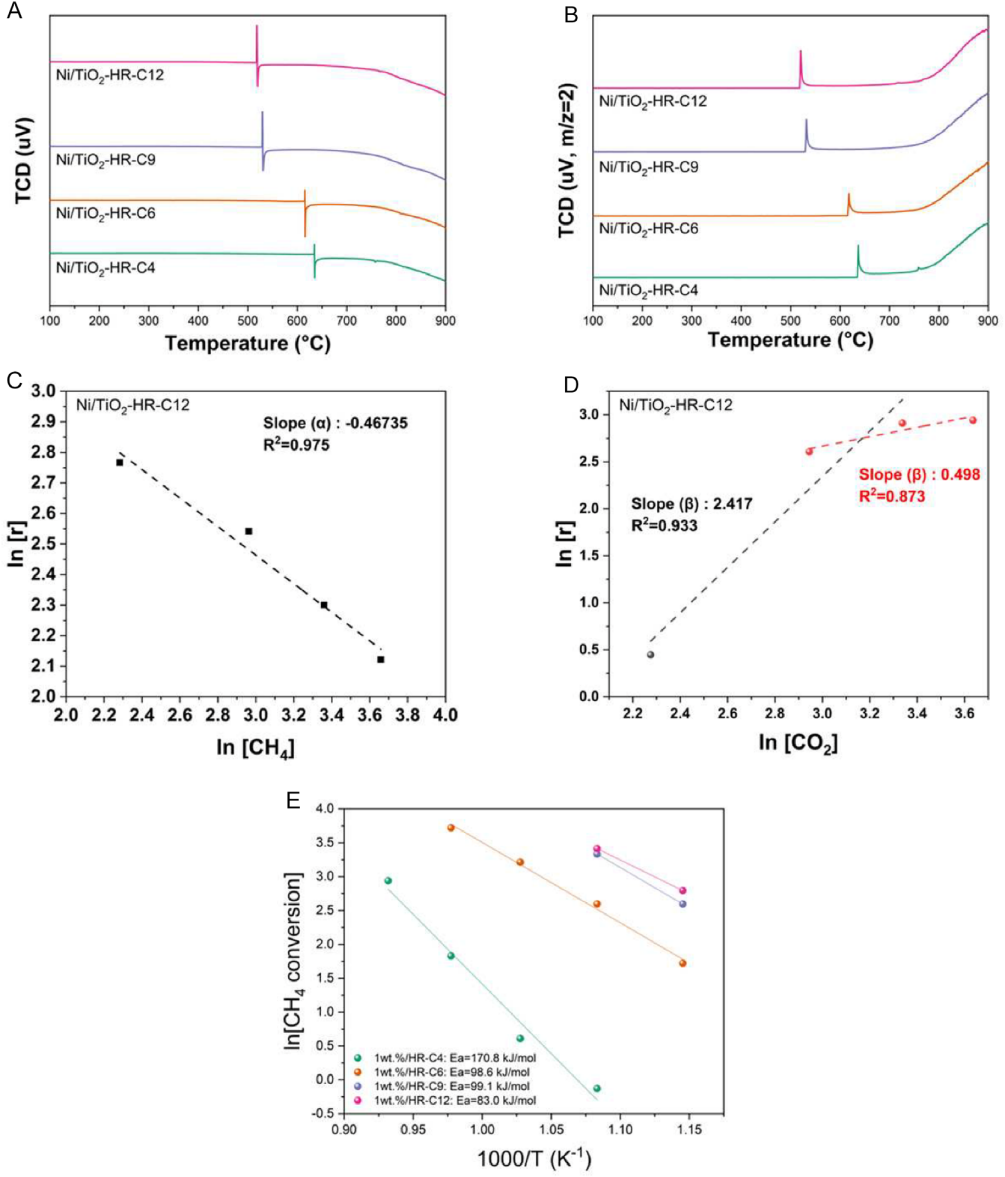
CH_4_ activation behavior and kinetic analysis of Ni/TiO_2_‐HR‐CX catalysts. (A) CH_4_‐TPSR profiles showing earlier CH_4_ activation for catalysts supported on high‐temperature‐calcined TiO_2_ (Ni/TiO_2_‐HR‐C9 and Ni/TiO_2_‐HR‐C12), compared with delayed activation for Ni/TiO_2_‐HR‐C4 and Ni/TiO_2_‐HR‐C6. (B) Corresponding H_2_ (*m*/*z* = 2) evolution signals, where Ni/TiO_2_‐HR‐C12 exhibits the highest integrated intensity, reflecting a greater number of accessible metallic Ni sites. (C) Reaction order with respect to CH_4_ for the most active catalyst (Ni/TiO_2_‐HR‐C12), indicating a negative value (−0.5), consistent with competitive adsorption between CH_4_ and CO_2_ on Ni sites. (D) Reaction order with respect to CO_2_ for Ni/TiO_2_‐HR‐C12, showing a transition from strongly positive behavior at low CO_2_ concentration to ≈0.5 under CO_2_‐rich conditions, suggesting a shift from CO_2_‐activation‐limited to mixed‐control kinetics. (E) Arrhenius plots used to determine the apparent activation energies (*E*_a,app_), demonstrating a significant reduction in the energy barrier for catalysts prepared with higher support calcination temperatures.

To clarify the mechanistic origin behind the DRM performance of the high‐temperature‐calcined catalysts, although their Ni particle sizes were similar, CH_4_‐TPSR and kinetic reaction‐order analysis were conducted (Figure 8A,B) [41]. CH_4_‐TPSR revealed a clear dependence of CH_4_ activation on the calcination temperature of the TiO_2_ support. For Ni/TiO_2_‐HR‐C9 and Ni/TiO_2_‐HR‐C12, the formation of H_2_ (*m*/*z* = 2) began at ∼500°C–550°C, indicating that CH_4_ activation occurred at a relatively low temperature. In contrast, Ni/TiO_2_‐HR‐C4 and Ni/TiO_2_‐HR‐C6 exhibited delayed H_2_ evolution, which appeared only at 600°C–650°C. Although Ni/TiO_2_‐HR‐C9 and Ni/TiO_2_‐HR‐C12 exhibited nearly identical onset temperatures, the integrated H_2_ signal was larger for Ni/TiO_2_‐HR‐C12, demonstrating that the high‐temperature‐calcined support provided a greater number of accessible metallic Ni sites.

Furthermore, to quantitatively evaluate the energy barrier for the DRM reaction, *E*_a,app_ (apparent activation energy) values were determined from Arrhenius plots (Figure 8E). The results showed a significant decrease in the energy barrier as the support calcination temperature increased: Ni/TiO_2_‐HR‐C4 (170.8 kJ/mol) > Ni/TiO_2_‐HR‐C6 (98.6 kJ/mol) ≈Ni/TiO_2_‐HR‐C9 (99.1 kJ/mol) > Ni/TiO_2_‐HR‐C12 (83.0 kJ/mol). This trend provides definitive evidence that the structural stabilization of the TiO_2_ support, which prevents Ni encapsulation and ensures high active site accessibility, directly lowers the apparent activation energy. Specifically, the marked reduction in *E*_a,app_ for Ni/TiO_2_‐HR‐C12 (83.0 kJ/mol) compared to Ni/TiO_2_‐HR‐C4 (170.8 kJ/mol) underscores that accessibility is the primary factor governing the catalytic efficiency in this system.

The kinetic response of the most active catalyst (Ni/TiO_2_‐HR‐C12) was further examined by determining the reaction orders with respect to CH_4_ and CO_2_. The total flow rate was maintained at 50 mL min^−1^, and the concentration of a single reactant was varied while the other was held constant. Conversions were restricted to 10%–20% to ensure quasi‐differential conditions (Figure 8C,D) [42]. The experimentally derived rate expressions are summarized as follows:

At low CO_2_ concentrations:

$$r=k{\left[{\mathrm{CH}}_{4}\right]}^{-0.5}\cdot {\left[{\mathrm{CO}}_{2}\right]}^{2.4}$$

At high CO_2_ concentrations:

$$r=k{\left[{\mathrm{CH}}_{4}\right]}^{-0.5}\cdot {\left[{\mathrm{CO}}_{2}\right]}^{0.5}$$

The reaction‐order analysis provides insight into the adsorption and activation behavior of CH_4_ and CO_2_ over Ni/TiO_2_‐HR‐C12. The negative reaction order for CH_4_ (∼−0.5) suggests that methane competes with CO_2_ for adsorption on Ni sites, which can suppress the formation of surface CO_*x*_ species under CH_4_‐rich conditions. In contrast, the strongly positive apparent reaction order for CO_2_ at low concentration (∼2.4) indicates that the reaction rate responds sensitively to the availability of CO_2_‐derived adsorbed species. As the CO_2_ concentration increases, the reaction order decreases to ∼0.5, implying that the kinetic influence of CO_2_ becomes less pronounced once a sufficient coverage of CO_2_‐derived intermediates is established [43, 44, 45]. These kinetic features are consistent with reported trends in Ni‐based DRM catalysts, where the balance between CH_4_ activation and CO_2_‐derived surface species varies depending on reactant composition and surface accessibility rather than a single fixed mechanistic step. In this system, the more pronounced kinetic response observed for CO_2_ correlates with improved Ni accessibility in the high‐temperature‐calcined supports, aligning with the TPSR and structural analysis.

## 3 Conclusion

This study demonstrates that the calcination temperature of TiO_2_ supports plays a decisive role in controlling pore evolution, Ni accessibility, and DRM catalytic behavior. Low‐temperature‐calcined TiO_2_ initially exhibited a hierarchical mesoporous‐macroporous structure, but the mesoporous framework collapsed upon high‐temperature Ni calcination, partially encapsulating Ni species and limiting catalytic accessibility. In contrast, supports calcined at 900°C or higher developed thermally stable macroporous or plate‐like morphologies that preserved a high fraction of surface‐exposed Ni species. Despite negligible differences in Ni particle size, Ni loading, and reducibility, the Ni/TiO_2_‐HR‐C9 and Ni/TiO_2_‐HR‐C12 catalysts showed markedly higher DRM activity and stability. These results confirm that Ni accessibility, rather than Ni crystal size, is the primary structure‐performance descriptor in this catalyst system. Furthermore, CO_2_‐TPD analysis confirmed that prestabilization at 1200°C, preserves reaction‐relevant strong basic sites, offering a chemical rationale for the catalyst superior resistance to coke‐induced deactivation. CH_4_‐TPSR and kinetic analysis reveal that the preserved accessibility of Ni sites directly governs the reactivity of the most active catalysts. Overall, this work provides insight into strategies for designing thermally robust oxide supports and demonstrates that maintaining accessible Ni species under high‐temperature conditions is more critical than maximizing surface area or mesoporosity.

## 4 Experimental Section/Methods

### 4.1 Reagents

Titanium isopropoxide (Ti(OiPr)_4_, 97.0%), nickel nitrate hexahydrate (Ni(NO_3_)_2_·6H_2_O, 97%) were purchased from Junsei chemical and used without further purification. All other reagents were of analytical grade and used as received.

### 4.2 Catalyst Preparation

TiO_2_ supports were synthesized via a modified hydrolytic precipitation method. Titanium isopropoxide (Ti(OiPr)_4_) was added dropwise into distilled water under vigorous stirring to induce controlled hydrolysis and precipitation. The resulting suspension was filtered, and the precipitates were thoroughly washed with distilled water to remove residual impurities. The obtained solids were dried and subsequently calcined at 400°C, 600°C, 900°C, and 1200°C for 2 h at a heating rate 5°C min^−1^ to prepare the TiO_2_ supports.

Ni‐loaded catalysts were prepared by the incipient wetness impregnation method. Each TiO_2_ support was impregnated with an aqueous Ni precursor solution to achieve a nominal Ni loading of 1 wt%. The impregnated samples were dried and calcined at 900°C for 2 h to obtain the final Ni/TiO_2_ catalysts.

### 4.3 Catalyst Characterization

Powder XRD patterns were collected on a PANalyical Empyrean diffractometer equipped with Cu Kα radiation (*λ *= 1.54 Å) operated at 40 kV and 50 mA over a 2*θ* range of 10°–80°.

N_2_ physisorption measurements were performed on a TRISTAR II 3020 analyzer (Micromeritics, Norcorss, USA) at −196°C to determine the surface area and pore size distribution. Prior to analysis, each sample was degassed under vacuum at 200°C for 3 h. Detailed pore structure analysis was further conducted using an AutoPore IV 9500 mercury intrusion porosimeter (Micromeritics, USA).

H_2_‐TPR experiments were carried out using a BELCAT II catalysts analyzer (MicrotracBEL, Japan). Samples were pretreated in pure Ar (50 mL min^−1^) at 200°C for 2 h and then cooled to 50°C. Reduction was performed in a 5% H_2_/Ar mixture (50 mL min^−1^) by heating from 50°C to 900°C at ramp rate of 10°C min^−1^.

The surface basicity of the catalysts was investigated by CO_2_‐TPD using BELCAT II instrument. The samples were pretreated under H_2_ flow at 800°C, cooled to 50°C, and saturated with CO_2_ gas. After purging with He, the desorption profiles were recorded from 50°C to 900°C.

XPS was conducted on a Thermo Scientific NEXSA spectrometer equipped with an Al Kα monochromatic source. Measurements were performed under ultrahigh‐vacuum conditions, and high‐resolution spectra were collected with a step size of 0.1 eV and averaged over 10 scans.

The morphology of porous TiO_2_ supports and Ni/TiO_2_ catalysts was examined using a JSM‐7900F field‐emission scanning electron microscope (FE‐SEM) (JEOL, Japan). Samples were coated with Pt to prevent charging, and structural variations in the supports were evaluated.

The metal content of the catalysts was determined using a Thermo Scientific iCAP 7400 DUO ICP‐OES instruments. For digestion, samples were treated in microwave pretreatment with 1 mL of HF and 3 mL of HNO_3_ at 280°C for 1 h.

TEM was performed using TECNAI F20 UT analytical electron microscope (Philips, Netherlands) at Korea Basic Science Institute. Crystal morphology and particle size were analyzed, and the average Ni particle size was determined by measuring at least 50 Ni particles using ImageJ software.

TGA was conducted using a TGA2 instrument (Mettler Toledo, Switzerland) under an air flow of 60 mL min^−1^. Samples were heated from 30°C to 900°C at a ramp rate of 10°C min^−1^ to quantify coke deposition.

### 4.4 Catalytic Performance Evaluation

The catalytic performance in DRM was evaluated in a 1/4‐inch quartz fixed‐bed reactor under ambient pressure using 300 mg of catalyst. Prior to the reaction, each catalyst was reduced in 100% H_2_ at 800°C for 2 h. The DRM reaction was then carried out at 700°C for 50 h with a CH_4_: CO_2_: N_2_ feed ratio of 1.0:1.1:1.0 to compensate for fluctuations in gas flow. The weight hourly space velocity (WHSV) was maintained at 10,000 mL g_cat_^−1 ^h^−1^.

The outlet gas composition was analyzed using a thermal conductivity detector (TCD) equipped Chromass 6500 GC system (Yong‐In, Korea). The CH_4_ and CO_2_ conversions and H_2_/CO ratio were calculated based on the molar flow rates (*F*_*x*_) according to the following equations:

$$X_{{\mathrm{CH}}_{4}}=\frac{{\left(F_{{\mathrm{CH}}_{4}}\right)}_{\mathrm{in}}-{\left(F_{{\mathrm{CH}}_{4}}\right)}_{\mathrm{out}}}{{\left(F_{{\mathrm{CH}}_{4}}\right)}_{\mathrm{in}}}$$

$$X_{{\mathrm{CO}}_{2}}=\frac{{\left(F_{{\mathrm{CO}}_{2}}\right)}_{\mathrm{in}}-{\left(F_{{\mathrm{CO}}_{2}}\right)}_{\mathrm{out}}}{{\left(F_{{\mathrm{CO}}_{2}}\right)}_{\mathrm{in}}}$$

$$H_{2}/CO\;ratio=\frac{{\left(F_{H_{2}}\right)}_{\mathrm{out}}}{{\left(F_{\mathrm{CO}}\right)}_{\mathrm{out}}}$$

## Supporting Information

Additional supporting information can be found online in the Supporting Information section.

## Author Contributions

**Sung‐Bin Choi**: conceptualization (lead); data curation (lead); formal analysis (lead); investigation (lead); methodology (lead); software (lead); visualization (lead); writing – original draft (lead); writing – review & editing (lead). **Ye‐** **Eun Jeon**: data curation (equal); formal analysis (supporting); investigation (supporting); software (supporting). **Da‐Bin Kang**: data curation (equal); visualization (equal). **Min‐Young Kim**: investigation (supporting); methodology (supporting). **Chang** **Hyun Ko**: conceptualization (lead); data curation (lead); funding acquisition (lead); project administration (lead); resources (lead); supervision (lead); validation (lead); writing – original draft (lead); writing – review & editing (lead).

## Funding

This study was supported by National Research Foundation of Korea (grant RS‐2025‐00557069), Korea Evaluation Institute of Industrial Technology (grant RS‐2023‐00265608).

## Conflicts of Interest

The authors declare no conflicts of interest.

## Supporting information

Supplementary Material

## Data Availability

The data that support the findings of this study are available from the corresponding author upon reasonable request.
