# Supplementary material for: Calcination‐Induced Pore Evolution in TiO2 Supports Governing Ni Accessibility for Dry Methane Reforming
Source: ChemistryOpen. 2026 Apr 20;15(5):e202600011. doi: 10.1002/open.202600011 (PMC13096576; doi:10.1002/open.202600011)
Supplement: Supplementary file 1 — Supplementary Material [file OPEN-15-e202600011-s001.pdf]

Supporting Information

Title

Calcination-Induced Pore Evolution in TiO_2 Supports Governing Ni Accessibility for Dry Methane Reforming

Sung-Bin Choi,[a] Ye-Eun Jeon,[a] Da-Bin Kang,[a] Min-Young Kim,[a] and Chang Hyun Ko*[a]

[a]
School of Chemical Engineering
Chonnam National University
Gwangju 61186
E-mail: chko@jnu.ac.kr

I. Supporting Figures and Tables

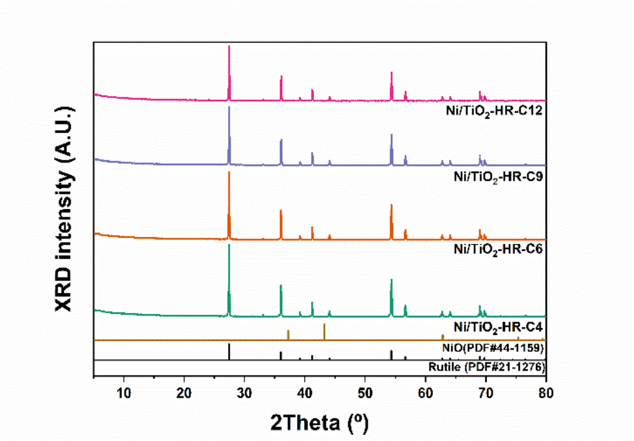

Figure S1. XRD patterns of Ni/TiO_2-HR-C*X* catalysts after Ni impregnation and 900 °C calcination. No distinct NiO phase was observed, indicating that the low Ni loading (≈ 1wt.%) and well dispersion prevented formation of detectable crystalline NiO domains.

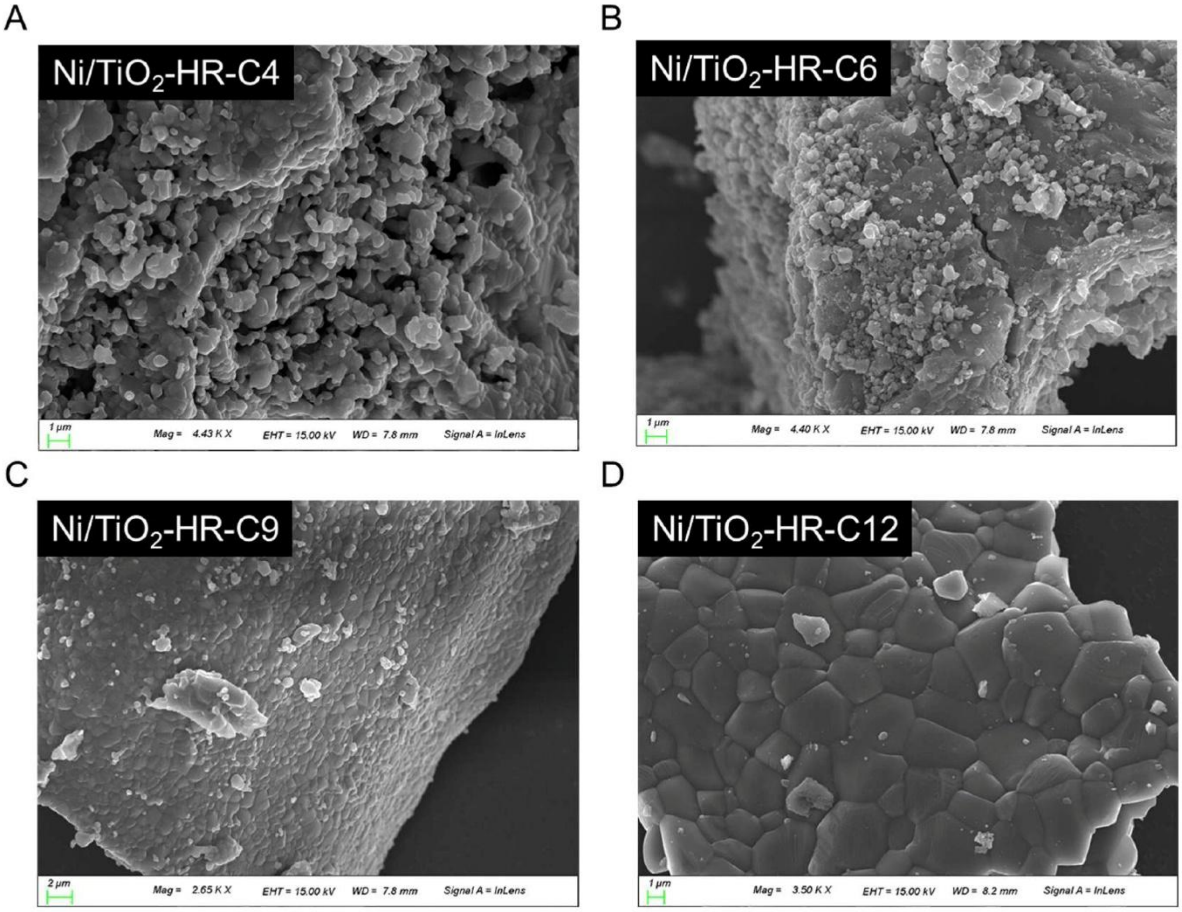

Figure S2. SEM images of Ni/TiO_2-HR-C*X* catalysts after Ni impregnation and calcination at 900 °C calcination. While macroscopic morphology can be compared across samples, the contrast between Ni and TiO_2 was insufficient to distinguish Ni particle distribution, and therefore the images are included for morphological reference only.

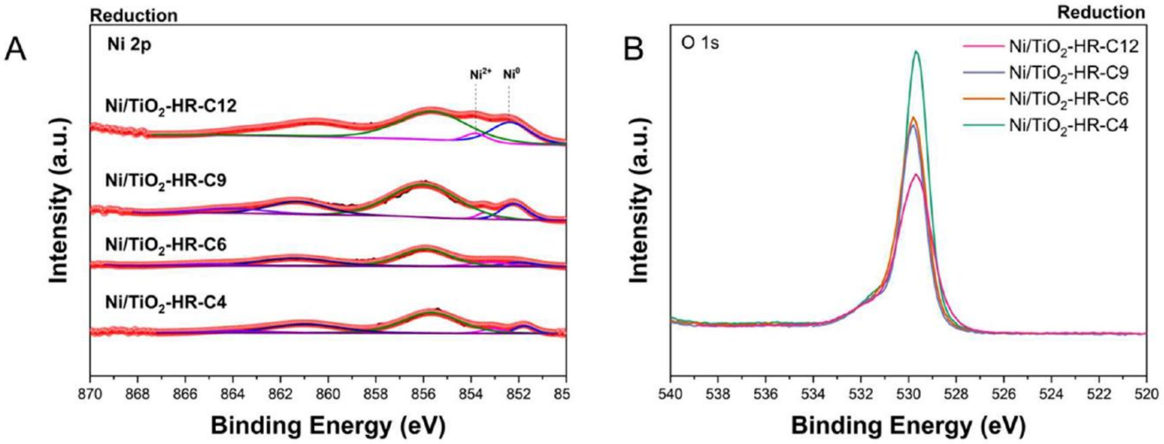

Figure S3. (A) Ni 2p spectra after H_2 reduction at 800 °C. The surface Ni signals progressively increase from Ni/TiO_2-HR-C4 to Ni/TiO_2-HR-C12, demonstrating higher surface exposure of Ni species on the high-temperature-calcined supports, consistent with improved catalytic performance. (B) O 1s XPS spectra showing a decrease in intensity with increasing calcination temperature, indicative of thermal densification.

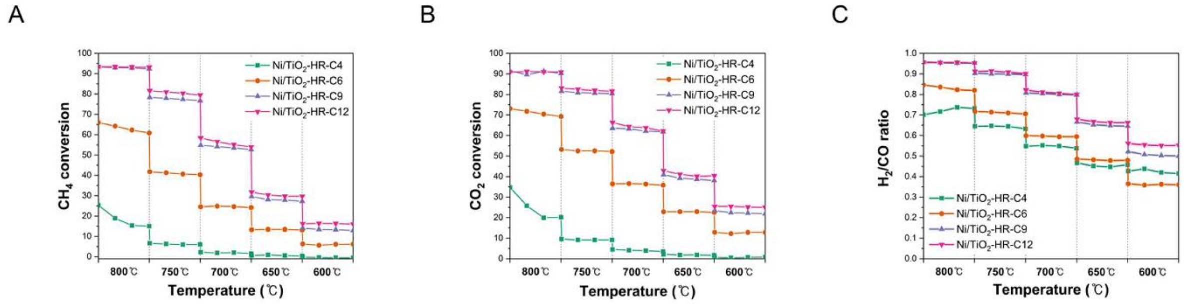

Figure S4. Temperature-dependent DRM performance of Ni/TiO_2-HR-C*X* catalysts: (A) CH_4 conversion, (B) CO_2 conversion, and (C) H_2/CO ratio recorded at each temperature step. For each condition, four consecutive points were acquired to ensure reproducibility. The averaged values from these measurement are reported in Figure 6.

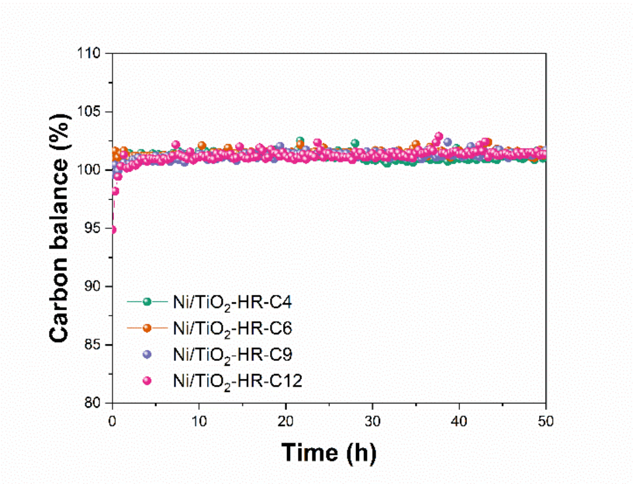

Figure S5. Carbon balance of Ni/TiO_2-HR-C*X* catalysts during DRM stability tests at 700 °C. The carbon balance for all catalysts remained within the range of 100 ± 2% throughout the 50-hour reaction period, confirming the high accuracy of the gas chromatography (GC) analysis and the reliability of the reported conversion and selectivity data.

Table S1. Average carbon balance for the investigated catalysts during the stability tests.

	Ni/TiO_2-HR-C4	Ni/TiO_2-HR-C6	Ni/TiO_2-HR-C9	Ni/TiO_2-HR-C12
Average Carbon Balance (%)	101.21	101.41	101.23	101.20
Standard Deviation	0.28	0.24	0.34	0.70
